# Correction to: Physiological insights of recent clinical diagnostic and therapeutic technologies for cardiovascular diseases

**DOI:** 10.1007/s12576-017-0578-0

**Published:** 2017-11-21

**Authors:** Kenji Shigemi, Soichiro Fuke, Dai Une, Keita Saku, Shuji Shimizu, Toru Kawada, Toshiaki Shishido, Kenji Sunagawa, Masaru Sugimachi

**Affiliations:** 1 Department of Anesthesiology and Reanimatology, University of Fukui Faculty of Medical Sciences, Fukui, Japan; 2 Department of Cardiology, Japanese Red Cross Okayama Hospital, Okayama, Japan; 3 Division of Cardiovascular Surgery, Yamato Seiwa Hospital, Yamato, Kanagawa Japan; 4 Department of Therapeutic Regulation of Cardiovascular Homeostasis, Center for Disruptive Cardiovascular Medicine, Kyushu University, Fukuoka, Japan; 5 Department of Cardiovascular Dynamics, National Cerebral and Cardiovascular Center, 5-7-1 Fujishiro-dai, Suita, Osaka 565-8565 Japan; 6 Department of Research Promotion, National Cerebral and Cardiovascular Center, Suita, Osaka Japan

## Correction to: J Physiol Sci (2017) 67:655–672 https://doi.org/10.1007/s12576-017-0554-8

The article Physiological insights of recent clinical diagnostic and therapeutic technologies for cardiovascular diseases, written by Kenji Shigemi, Soichiro Fuke, Dai Une, Keita Saku, Shuji Shimizu, Toru Kawada, Toshiaki Shishido, Kenji Sunagawa and Masaru Sugimachi, was originally published Online First without open access. After publication in volume 67, issue 6, page 655-672 the author decided to opt for Open Choice and to make the article an open access publication. Therefore, the copyright of the article has been changed to © The Author(s) 2017 and the article is forthwith distributed under the terms of the Creative Commons Attribution 4.0 International License (http://creativecommons.org/licenses/by/4.0/), which permits use, duplication, adaptation, distribution and reproduction in any medium or format, as long as you give appropriate credit to the original author(s) and the source, provide a link to the Creative Commons license, and indicate if changes were made.

